# Myocarditis and Myocardial Injury in Long COVID Syndrome: A Comprehensive Review of the Literature

**DOI:** 10.7759/cureus.42444

**Published:** 2023-07-25

**Authors:** Sai Sri Hari Paruchuri, Umm E Farwa, Shaista Jabeen, Shreyansh Pamecha, Zoofi Shan, Ritika Parekh, Mohit Lakkimsetti, Eman Alamin, Vagisha Sharma, Salar Haider, Javeria Khan, Waleed Razzaq

**Affiliations:** 1 Internal Medicine, Dr. Pinnamaneni Siddhartha Institute of Medical Sciences and Research Foundation, Vijayawada, IND; 2 Emergency Medicine, Jinnah Sindh Medical University, Karachi, PAK; 3 Medicine, Pakistan Air Force (PAF) Hospital, Islamabad, PAK; 4 Internal Medicine, All India Institute of Medical Sciences, Raipur, Raipur, IND; 5 Cardiology, Hero DMC (Dayanand Medical College) Heart Institute, Ludhiana, IND; 6 Community Health, K. J. (Karamshibhai Jethabhai) Somaiya Medical College and Research Centre, Mumbai, Mumbai, IND; 7 Internal Medicine, Mamata Medical College, Khammam, IND; 8 Community Health, University of Medical Sciences and Technology, Khartoum, SDN; 9 College of Medicine, Vardhaman Mahavir Medical College and Safdarjung Hospital, New Delhi, IND; 10 Physiology, Shifa College of Medicine, Islamabad, PAK; 11 Adult Cardiology, National Institute of Cardiovascular Diseases, Karachi, PAK; 12 Internal Medicine, Services Hospital Lahore, Lahore, PAK

**Keywords:** cardiac outcomes, sars-cov-2, covid and cardiovascular complications, cardiovascular side effects, covid-induced myocarditis, acute myocardial injury, covid 19, covid and myocarditis

## Abstract

The repercussions of coronavirus disease 2019 (COVID-19) have been devastating on a global scale. Long COVID, which affects patients for weeks or even months after their initial infection, is not limited to individuals with severe symptoms and can affect people of all ages. The condition can impact various physiological systems, leading to chronic health conditions and long-term disabilities that present significant challenges for healthcare systems worldwide. This review explores the link between long COVID and cardiovascular complications such as myocardial injury and myocarditis. It also highlights the prevalence of these complications and identifies risk factors for their development in long COVID patients. Myocardial injury occurs due to direct cellular damage and T-cell-mediated cytotoxicity resulting in elevated cardiac biomarkers. Diagnostic techniques like electrocardiogram, troponin level testing, and magnetic resonance imaging can help identify myocarditis, but endomyocardial biopsy is considered the gold-standard diagnostic technique. Guideline-directed medical therapy is recommended for COVID-19 myocarditis patients for better prognosis while being monitored under comprehensive care management approaches. Therefore, it's critical to develop effective screening techniques specifically for vulnerable populations while conducting further research that addresses the effects of long COVID on society's physical health.

## Introduction and background

The outbreak of severe acute respiratory syndrome coronavirus 2 (SARS-CoV-2), which is a viral infection that results in coronavirus disease 2019 (COVID-19), began in Wuhan, China, in December 2019. Since then, this virus has had severe consequences for various organ systems within the human body. Despite numerous attempts by governments worldwide to reduce the spread of COVID-19, it has continued to ravage populations globally and has resulted in a devastating pandemic. The COVID-19 pandemic has had a significant impact on humanity and caused millions of deaths worldwide. As of June 1st, 2023, data from the World Health Organization (WHO) reveals that over 761 million cases of COVID-19 have been reported globally, resulting in nearly 6.9 million fatalities [1]. It is worth noting that despite the alarming statistics, the mortality rate for COVID-19 remains less than 1%, and this can be attributed mainly to severe acute respiratory syndrome and multiple organ dysfunction [2]. It is essential to recognize that, among other potential complications related to COVID-19, myocarditis is particularly concerning as it causes inflammation of the heart muscle, leading to life-threatening conditions such as acute non-ischemic myocardial injury, systolic heart failure, cardiogenic shock, and arrhythmias. Myocarditis can occur as a result of direct viral damage or an overly reactive immune response [3]. Indeed, some individuals infected with COVID-19 have even succumbed to this condition [4]. Given these serious findings, there is an urgent need for continuous monitoring and research regarding COVID-19's clinical manifestations to ensure optimal patient care strategies are implemented worldwide.

Myocarditis is usually caused by various factors, such as viral and bacterial infections. Myocyte destruction occurs due to myocarditis, which can ultimately result in cardiomyopathy, heart failure, and sudden cardiac death (due to arrhythmias mainly) [4]. 

Those who have contracted the virus may present with a broad spectrum of symptoms that vary in their severity. The manifestation of these symptoms may range from being inconspicuous or absent to being serious and multifaceted. Some individuals may experience only minor symptoms like tiredness and breathing difficulties, whereas others may suffer from more pronounced ones such as chest pain or tightness while exerting themselves physically. It is worth noting that, in certain cases, the condition can take a critical turn and quickly worsen, causing manifestations like elevated heart rate and acute heart failure, which can ultimately lead to cardiogenic shock [2]. Currently, the dominant manifestation of the virus is characterized by rapid and severe inflammation of the heart muscle, commonly referred to as fulminant myocarditis. The condition results in heart failure and impaired functioning of the ventricles, typically manifesting within a period of two to three weeks after contracting the virus. It is worth noting that fulminant myocarditis can often be mistaken for sepsis and display indicators like fever, low pulse pressure, cold extremities, and sinus tachycardia [2].

Due to limitations in diagnostic techniques, myocarditis is frequently overlooked during the COVID-19 diagnosis. However, several testing methods have been established to confirm the presence of the disease. These include an electrocardiogram with ST elevation, laboratory testing including serum troponin levels, and imaging like cardiac magnetic resonance to confirm the diagnosis [5]. For a definite diagnosis of myocarditis, an endomyocardial biopsy (EMB) may be necessary, which is considered the gold standard diagnostic method [5]. 

People who are believed to have been infected with SARS-CoV-2, the virus responsible for COVID-19, may be diagnosed with long COVID if their symptoms persist for at least two months after the onset of their initial symptoms and occur within roughly three months of their first infection. It is important to note that this diagnosis can only be given when other conditions have been ruled out as potential causes of the symptoms. Long COVID is a condition that has become increasingly recognized as more people experience lingering effects from COVID-19 even after they have recovered from the acute phase of the illness. These persistent symptoms can range from fatigue and brain fog to difficulty breathing and chest pain, among others. Long COVID researchers will continue to learn more about how long it lasts, who is most likely to develop it, and how to treat those who have it [6].

Long COVID may have an impact on numerous physiological systems, including the respiratory, cardiovascular, gastrointestinal, renal, neurological, and musculoskeletal [7]. The most important cardiovascular symptoms are chest pain, palpitations, fainting, shortness of breath, tiredness, and swelling in the lower limbs [6,7]. The most common heart problems are dysrhythmias, cardiac injury, ischemic heart disease, venous thromboembolism, dysautonomia, heart failure, cardiomyopathy, and last but not least, inflammatory heart disorders like pericarditis and myocarditis [7,8]. In this review, we shall explain in detail the myocarditis complication of the long COVID syndrome.

## Review

Rationale 

It is of utmost importance to comprehend the correlation between long COVID and myocarditis or myocardial injury, as it plays a critical role in the formulation of efficacious diagnostic and treatment methodologies. Given the paucity of research on this matter, a complete analysis of the existing literature can prove invaluable in identifying areas where information is lacking and where further investigations are warranted. By conducting a comprehensive review, one can identify potential avenues for scientific inquiry that will shed light on this crucial area of study.

Epidemiology and prevalence of long COVID syndrome

Amidst the COVID-19 pandemic, patients have reported experiencing enduring and incapacitating symptoms, including but not limited to cognitive dysfunction, fatigue, and respiratory distress. The aforementioned concerns were extensively deliberated across various social media channels. It is noteworthy that individuals who undergo severe illness, hospitalization, or intensive treatment may develop a condition known as post-intensive care syndrome (PICS), which can lead to diminished functionality for a duration of up to one year following discharge. The aforementioned phenomenon poses a challenge in discerning the symptoms that are linked to post-COVID-19 conditions like long COVID, long-haul COVID, post-acute sequelae of SARS-CoV-2 infection (PASC), and chronic COVID [9], as these conditions require a considerable amount of time to manifest. The term “long COVID” has been popularized by patients on social media platforms such as Twitter (Twitter, Inc., San Francisco, California, United States) and Reddit (Reddit Inc., San Francisco, California, United States), as medical experts have yet to establish an official diagnosis for the persistent symptoms of COVID-19. Therefore, these virtual discussion boards have served as a medium for individuals to exchange their personal accounts and establish relationships with others who are encountering comparable obstacles in their journey toward rehabilitation [10].

According to the World Health Organization (WHO), post-COVID-19 condition is defined as symptoms that are inexplicable by any alternative diagnosis, last for at least two months, impact day-to-day functioning, and occur three months from the onset of COVID-19 [11]. Many cohort studies with questionnaires have been used to follow up on patients fitting this criteria. One cohort of 234 participants 3-9 months after their initial acute illness was surveyed to find that 30% of them reported symptoms six months later and 30% also reported a worsened quality of life [12]. Many retrospective cohorts of this type were conducted early in the pandemic. Another cohort study performed an anonymous online survey of 3,762 participants from 56 different countries. It was found that 55.5% of participants experienced cognitive dysfunction in their seventh month after illness, and 45.2% reported the need to reduce their work schedule. The study collected a total of 66 reported symptoms that were traced over seven months [13].

Lung diffusion capacity and health-related quality of life (HRQoL) were also examined by a prospective cohort performed on 101 patients six weeks post discharge. Seventy-two percent of patients showed a diffusing capacity of the lungs for carbon monoxide (DLCO) <80% predicted value, indicating a limitation of lung diffusion. Even non-critical COVID-19 pneumonia survivors report decreased HRQoL [14]. Another cohort measured fatigue in study participants using the Chalder Fatigue Score (CFQ-11) and found that over 50% of participants reported fatigue (not associated with initial disease severity) approximately 10 weeks after their initial illness [15]. A similar cohort found 34.8% of their patients to have at least one symptom seven months after symptom onset. It was observed that anosmia and/or diarrhea during the initial acute illness were associated with a higher chance of incurring longer-term symptoms [16]. Cardiac outcomes were also observed in a cohort that performed cardiovascular magnetic resonance (CMR) imaging on 100 patients at a median time interval of 71 days after their diagnosis; 60% of their patients showed ongoing myocardial inflammation [17]. A cohort study performed in a single hospital found additional symptoms such as alopecia in 28.6% of their participants, along with a small number of patients reporting a recent diagnosis of hypertension since their initial acute illness [18].

Long COVID can present with a constellation of symptoms with variable onset and intensity, making it difficult to accurately estimate its prevalence. The diagnosis will require a robust patient history and follow-up. A prospective cohort performed as a telephone survey sampled 1250 post-COVID-19 discharge patients, of whom 488 responded to the telephone survey. Of the respondents, 159 (32.6%) reported cardiopulmonary symptoms 60 days after being discharged. The authors of this cohort write about the limitations, as half of their sample did not complete their follow-up. It was also noted that although most patients went to a primary care physician (PCP) post discharge, 20% of the patients had no primary care follow-up visit within two months post discharge [19]. One specific cohort demonstrated that there was no significant difference in the risk of any feature of long COVID between unvaccinated and vaccinated patients post acute illness [20]. An additional cohort surveyed by trained physicians approximately 111 days after discharge compared two patient groups: patients requiring ICU care and those managed in the hospital wards. The study concluded that the appearance of persistent symptoms in both groups was similar and that no significant difference in HRQoL exists between the two patient groups [21].

In 2020, Italy set up a specialized outpatient clinic to monitor the recovery of discharged patients and captured a sample size of 31,845 confirmed cases. The patients were all discharged following the WHO criteria for discontinuation of quarantine, and 71.4% of the cases sampled had symptoms 60 days after discharge, while 87.4% reported experiencing at least one symptom. The authors of the study, Carfì et al., note that there may be a bias due to patient self-reporting and a lack of details on the severity of symptoms [22]. According to a study by the United Kingdom (UK) Office for National Statistics, one in five participants who tested positive for COVID-19 had symptoms that lasted for five weeks or longer. Approximately one in 10 participants exhibited symptoms for 12 weeks or longer [23]. The ONS also released a census of 1.9 million UK residents who self-reported symptoms beyond four weeks from their first confirmed or suspected COVID-19 infection [24]. The UK government published its latest statistics on an online webpage where they have notated 19,224,278 total first infections, with 1.53 million cases of reinfection [25]. This would correlate to approximately 10% of confirmed cases presenting with symptoms of long COVID. 

Without more precise ways of diagnosing long COVID, it will remain difficult to estimate its prevalence. Some studies have demonstrated that patients with long COVID have an elevated humoral antibody response to SARS-CoV-2 viral pathogens with increased levels of interleukin-8 (IL-8), chemokine ligands 4 (CCL4), and other soluble immune mediators. Cross-reactivity with a herpesvirus was observed [26]. The future development of objective biomarkers to diagnose long COVID is highly necessary. According to the WHO global COVID-19 dashboard, as of April 2023, there have been 762,201,169 cumulative cases of COVID-19 in the world so far [27]. Even the above-suggested 10% prevalence would prove to be a huge number of worldwide patients afflicted.

Cardiovascular complications in long COVID syndrome

The available data on long COVID syndrome is currently limited. Nonetheless, we have conducted a thorough literature review, which has documented evidence of myocarditis or myocardial injury in patients with COVID-19. Several studies have suggested that individuals with long COVID syndrome may be at increased risk for developing cardiovascular diseases such as myocarditis, arrhythmias, and heart failure [28,29]. 

A study reported that cardiac damage, shock, and arrhythmia were prevalent among hospitalized patients. Specifically, the incidence of acute cardiac damage, shock, and arrhythmia was 7.2%, 8.7%, and 16.7%, respectively, among the cohort comprising 138 hospitalized patients [28]. It is noteworthy that these occurrences were found to be more frequent in patients who required intensive care. Additionally, a study conducted by the United States Department of Veterans Affairs Health Services revealed an increased incidence of cardiovascular and metabolic diseases in over 73,000 non-hospitalized COVID-19 patients who experienced death within a month after contracting the virus. Furthermore, a UK-based study on an extensive cohort involving nearly 48,000 hospitalized COVID-19 patients showed they had a three-time higher risk for cardiovascular events four months after discharge compared to non-hospitalized individuals. These reports highlight how coronaviruses may not only affect a person's respiratory system but also pose significant implications for their cardiovascular health. The study underscores the need to further evaluate and undertake appropriate treatment protocols to mitigate its effects on heart health. Notably, both studies suggest that men are at higher risk for such post-long COVID-related complications related to cardio events [29].

As per a comprehensive literature review on the aftermaths of long COVID syndrome, clinical outcomes, and plausible molecular mechanisms, it has been found that one-fourth of patients who were hospitalized due to COVID-19 suffered from myocardial injury, which increased their likelihood of mortality. Furthermore, post-mortem histological examinations of COVID-19 patients revealed myocardial hypertrophy in a staggering 92.9% of cases, mild to severe coronary artery atherosclerosis in all cases examined (100%), and localized myocardial fibrosis in 21.4% of cases [30].

A review concluded that the persistent nature of cardiovascular symptoms in long COVID patients is a significant concern. This is highlighted by current evidence showing ongoing heart-related issues such as pain, rapid heart rate, and breathlessness, even after acute symptoms have resolved. The evidence of myocardial injury, persistent elevation of troponin levels, myocarditis-like scarring, and late gadolinium enhancement in a non-ischaemic myocarditis-like pattern further supports the seriousness of the impact on the cardiovascular system [31].

The findings of a research investigation involving 26 collegiate athletes who had experienced mild or asymptomatic COVID-19 infection indicated that 15% of the subjects were diagnosed with myocarditis through the use of cardiac magnetic resonance imaging (MRI), while 30.8% displayed indications of previous myocardial damage [32]. A multidisciplinary clinic report showed that tachycardia and palpitations were persistent in 25-50% of patients 12 weeks after COVID-19 and in 9% of patients six months after COVID-19 [33].

In conclusion, there is significant evidence to suggest a persistent and impactful effect of COVID-19 on cardiovascular health. These effects include prolonged chest pain, palpitations, and dyspnea, as well as more severe complications like arrhythmias, pericarditis, and myocardial damage. Cardiac involvement is seen in a large proportion of patients post COVID-19, with some experiencing cardiac inflammation, regional scarring, and pericardial enhancement. This highlights the importance of long-term cardiovascular monitoring and care in COVID-19 recovery [31-52] (Table 1).

**Table 1:** Cardiovascular complications in association with long COVID syndrome COVID-19: coronavirus disease 2019; NIS: National (Nationwide) Inpatient Sample; VT: ventricular tachycardia; EF: ejection fraction

Source	Title	Country	Type of publication	Main findings	Prognosis of the patient
Szarpak et al., 2022 [4]	Myocarditis: A Complication of COVID-19 and Long-COVID-19 Syndrome as a Serious Threat in Modern Cardiology	Poland, Italy, United States	Letter (used for literature review only)	Complication of COVID-19 in light of myocarditis discussed	Prognosis is such that if individuals are vaccinated then that only protects a patient from getting the virus and developing its worst complications
Mele et al., 2021 [5]	Myocarditis in COVID-19 Patients: Current Problems	Italy	Compiled information from case report and case series	Mainly identified by chest pain, ST elevation, increased troponin	Good prognosis, favorable outcome in hospitalized patients and discharged. But those with cardiogenic shock died.
Theetha Kariyanna et al., 2020 [37]	A Systematic Review of COVID-19 and myocarditis	United States	Systematic review	Over the age of 50; Both genders equally affected, most common presenting symptoms included dyspnea, chest pain, coughing, fever.	No significant difference in prognosis with virus + or virus - patients
Blankstein and Chandrashekhar, 2021 [38]	New Insights on COVID-19 and Heart	Germany	Cohort	Chest pain further seen if positional or differs with inspiration	
Annie et al., 2022 [39]	Association with Myocarditis and Mortality in COVID-19 Patients in a Large Registry	United States vs Outside the United States	Cohort		Poor prognosis of cardiac involvement in COVID-19 patients Evident association between covid-19 and myocarditis mortality rates, especially those with comorbidity.
Agdamag et al., 2020 [3]	Update on COVID-19 Myocarditis	United States	Review	Fatigue, fever, chills, cough, git complications (young males more susceptible)	Pre-existing cardiac conditions and elevated troponin = worst prognosis high mortality, longer hospital stay and need for ventilation
Siripanthong et al., 2020 [2]	Recognizing Covid-19-related Myocarditis: the Possible Pathophysiology and Proposed Guideline for Diagnosis and Management	United Kingdom, Pennsylvania, Florida, Minnesota	Review	Fatigue, dyspnea, chest pain (Article discusses differentials also)	
Sattar et al., 2023 [41]	In-hospital Outcomes of Covid-19 Associated Myocarditis (From a Nationwide Inpatient Sample Database Study)	United States NIS databases	Cohort (COVID-19 with myocarditis and COVID-19 without myocarditis)		Higher prevalence of COVID-19 myocarditis in diabetes, chronic kidney failure, liver disease
Beşler and Arslan, 2020 [42]	Acute Myocarditis associated with COVID-19 Infection	Turkey	Case report		Febrile sensation and chest pain
Ramli et al., 2023 [43]	Post COVID-19 With Myocarditis: A Case Report	Malaysia	Case Report	A 44-year-old woman, gravida 7 para 6, at 32 weeks with complaints of palpitations, pre-syncopal attack, and tachycardia, had VT on the ECG and an EF of 60%.	She became asymptomatic with low-dose prednisolone.
Munoz et al., 2021 [44]	Cardiac Screening in a Young Adult Male Leading to Discovery of Post-COVID Myocarditis with Asymptomatic Large Apical Left Ventricular Thrombus	United States	Case Report	An 18-year-old individual who plays football experienced a three-day fever, myalgias, nausea, and vomiting. During the standard clearance process for the game, an increase in conventional troponin-I was detected.	He was diagnosed with COVID-19-related myocarditis, which led to a decreased ejection fraction and resulted in ventricular thrombus formation.
Pavon et al., 2020 [45]	First Documentation of Persistent SARS-Cov-2 Infection Presenting With Late Acute Severe Myocarditis	Switzerland	Case Report	A 64-year-old man presented with chest pain and dyspnea six weeks after being diagnosed with COVID-19 infection.	The patient developed severe cardiac involvement as seen on cardiac magnetic resonance imaging during the six-week asymptomatic period.
Rivera-Morales et al., 2020 [46]	Acute Myo-pericarditis in the Post COVID-19 Recovery Phase	United States	Case Report	A 73-year-old man presented to the ED with chest pain and dyspnea.	He was diagnosed with acute myopericarditis after noting the characteristic ECG findings like Spodick's sign.
Raman et al., 2022 [7]	Long COVID: Post-acute Sequelae of COVID-19 With a Cardiovascular Focus	United Kingdom	Review Article	The review article discusses the meaning of long COVID along with its epidemiology, pathophysiology, and management of cardiopulmonary manifestations.	Long COVID is a major health issue; however, even with limited knowledge about the same, it is vital to conduct screening tests to especially look for different organ system involvement and to help patients who are anxious about the same.
Khetpal et al., 2013 [48]	Long-term Cardiovascular Manifestations and Complications of Covid-19: Spectrum and Approach to Diagnosis and Management	United States	Review	This review discusses the long-term cardiovascular complications of COVID-19.	In most cases, the cardiac manifestations detected among the COVID-19 survivors were dysrhythmias, inflammatory diseases, ischemic diseases, heart failure, and dysautonomia.
Kotecha et al., 2021 [49]	Patterns of Myocardial Injury in Recovered Troponin-positive Covid-19 Patients Assessed by Cardiovascular Magnetic Resonance	United Kingdom	Original Research	148 patients who had a severe COVID-19 infection with elevated troponin levels were studied using cardiac magnetic resonance imaging at a median of 68 days from discharge.	Out of the 148 patients, 54% had late gadolinium enhancement and/or ischemia, a myocarditis-like scar in 26%, an infarction and/or ischemia in 22%, and a dual pathology in 2%.
Breitbart et al., 2021 [50]	Clinical and Cardiac Magnetic Resonance Findings in Post-Covid Patients Referred for Suspected Myocarditis	Germany	Original Paper	Using ECG, cardiac biomarkers, an echocardiograph, and cardiac magnetic resonance imaging, 56 post-COVID patients who still had symptoms like chest pain or discomfort, shortness of breath, and intolerance to activity were studied.	Through a thorough diagnostic process, only 2% of the patients were found to have myocarditis.
Huang et al., 2020 [51]	Cardiac Involvement in Patients Recovered From Covid-2019 Identified Using Magnetic Resonance Imaging	China	Original Research	A research investigation was conducted on a group of 26 individuals who recuperated from COVID-19, encountered cardiovascular complications and underwent cardiac magnetic resonance imaging.	Cardiac magnetic resonance imaging showed myocardial edema and late gadolinium enhancement in 58% of the patients.
Puntmann et al., 2019 [17]	Outcomes of Cardiovascular Magnetic Resonance Imaging in Patients Recently Recovered From Coronavirus Disease 2019 (COVID-19)	Frankfurt	Prospective observational cohort study	The investigation involved a group of one hundred individuals who had recuperated from COVID-19 and were selected from the University Hospital Frankfurt COVID-19 database during the period of April to June 2020.	Cardiac magnetic resonance imaging showed that the heart was involved in 78% of patients and that 60% of patients had ongoing inflammation of the myocardium. This was independent of the pre-existing conditions of the patients and the severity of COVID-19.

Pathogenesis 

Pathophysiological indications of myocardial injury and myocarditis have been observed in patients diagnosed with COVID-19. COVID-19 is believed to cause troponin elevation, which may result from physiological stress on the heart, hypoxia, or direct harm to the myocardium. Research has shown that up to 17% of hospitalized COVID-19 patients and 22-31% of those in the ICU could suffer from elevated troponin levels and myocardial injury [53]. According to Esfandiarei et al. and McManus et al., viral myocarditis' pathophysiology entails two mechanisms: cellular harm and T-lymphocyte-mediated cytotoxicity, which can be escalated by cytokine storm syndrome [2].

In COVID-19 patients, heightened levels of cardiac biomarkers, for example, cardiac troponin, have been discovered to indicate cardiac injury. This is closely associated with more severe disease progression and has the potential to predict hospitalization in the ICU and loss of life. The systemic inflammation and cytokine storm typical of COVID-19 have been linked to an increase in levels of inflammatory biomarkers such as IL-6 and C-reactive protein (CRP) [54]. These mediators have the potential to cause myocardial depression in cases where there is systemic hyper-inflammation, such as sepsis. Additionally, it has been observed that direct viral invasion of the myocardium can be boosted by an increased expression of human angiotensin converting enzyme-2 (ACE2) receptor in the myocardium due to the binding between spike proteins of COVID-19 and numerous viral particles entering cardiac fibers [55].

Numerous studies have demonstrated that human coronaviruses are linked to myocarditis in individuals of all ages. Once inside the human body, COVID-19 attaches itself to the ACE2 membrane protein by means of its spike protein. ACE2 is found in ciliated columnar epithelial cells of the respiratory tract, type II pneumocytes, and cardiomyocytes. This suggests that COVID-19 may pose a risk to human heart health, particularly in cases where ACE2 expression has been elevated due to heart failure. Nevertheless, it should be noted that viral receptor presence alone does not always serve as an accurate predictor of viral tropism [2].

The molecular pathophysiology of COVID-19 myocarditis involves the virus binding to ACE2 through its spike protein, which requires cleavage at the S1/S2 and S2 sites. A serine protein named transmembrane protease, serine 2 (TMPRSS2) mediates this cleavage. After entering human cells, COVID-19 may impede stress granule formation via its accessory protein, thus enabling replication and damage. Hepatocyte growth factor (HGF) produced by the heart can prime T-lymphocytes for viral antigens and cardiotropism, leading to CD8+ T-lymphocyte migration to cardiomyocytes and myocardial inflammation from cell-mediated cytotoxicity. The cytokine storm syndrome further amplifies T-lymphocyte activation, resulting in additional cytokine release and immune activation with subsequent myocardial injury [2].

Risk factors of myocarditis in patients with long COVID syndrome

There is a dearth of research on the risk factors associated with myocarditis in patients with long COVID syndrome [56]. However, some studies suggest that certain factors may increase the risk of myocarditis or myocardial injury in these patients.

**Figure 1:**
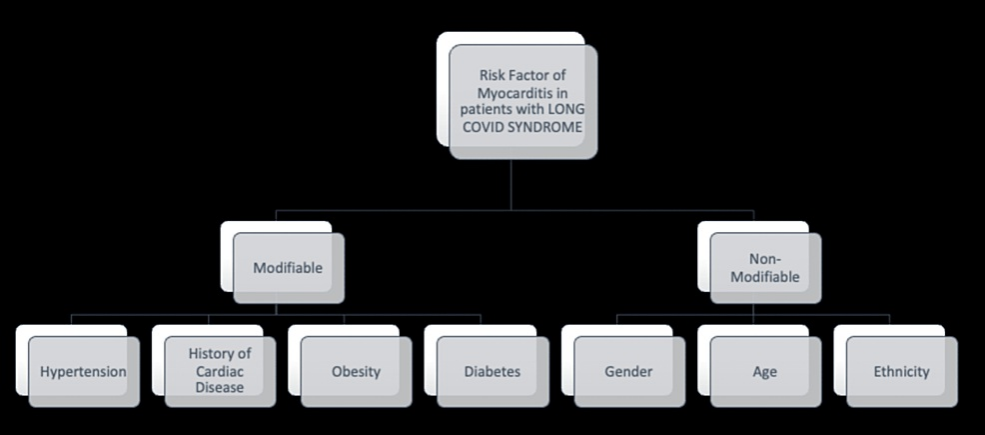
Most common risk factors of myocarditis in patients with long COVID syndrome, although by no means exhaustive COVID: coronavirus disease

Although the exact relationship between risk factors and post-COVID-19-syndrome-associated myocarditis has not yet been discovered, most patients who showed signs and symptoms of myocarditis had a past medical history of hypertension, obesity, diabetes mellitus [57], chronic kidney disease, chronic obstructive pulmonary disease, asthma, chronic liver disease, cerebral vascular accidents, coronary artery disease, and structural heart disease [58]. Some of the common risk factors of myocarditis in patients with long COVID syndrome have been shown in Figure 1.

Studies also report that most patients did not have a previously identified comorbid condition (50%); hypertension was the most predominant comorbidity in those patients with a past medical history (33%) [58,59]. Certain nonmodifiable factors have been determined through multiple studies, for instance, age and ethnicity. Many studies report that the male gender is more susceptible to developing post-COVID-19 myocarditis; two age groups were more prevalent: 12-17 years and men above 50 [60,61]. A study has also found that the incidence is higher in Asians, African Americans, Native Americans, Hispanics, and minority ethnic groups [62].

Exercise has reportedly been shown to exacerbate cardiac dysfunction in people who experience post-COVID myocarditis. Accelerated viral replication, more severe inflammation, and cellular death are thought to be the causes of this dysfunction. Being an athlete was probably a contributing aspect as well [62, 63].

Clinical presentation of myocarditis in patients with long COVID syndrome

Long COVID syndrome encompasses a wide range of symptoms that persist for more than two months after the initial COVID-19 infection and cannot be explained by any other diagnosis. These symptoms include, but are not limited to, prolonged fatigue, sensory deficits, neurocognitive deficits, and cardiovascular manifestations such as myocarditis and arrhythmias. Common cardiovascular symptoms during this period include chest pain, palpitations, shortness of breath with exertion, presyncope, fatigue, and pedal edema. Despite these notable cardiovascular effects associated with long COVID syndrome, the underlying causes remain unclear.

Long COVID syndrome significantly increases the risk of cardiovascular complications, underscoring the need for careful cardiac monitoring in COVID-19 patients. However, more extensive prospective studies are required for more precise estimates [64]. One report described the first instance of acute cardiac injury directly associated with myocardial localization of SARS-CoV-2. The patient had flu-like prodrome which swiftly degenerated into respiratory distress, hypotension, and cardiogenic shock [65]. The sufferer complained of exacerbating dyspnea, a constant cough, and fatigue for four days. The nasopharyngeal swab was conclusive for COVID-19 on the real-time reverse transcriptase-polymerase chain reaction (RT-PCR) assay. In three hours, LVEF dropped from 34% to 25%. According to convention, in non-ischaemic cardiogenic shock, an endomyocardial biopsy was done, which revealed viral particles in structurally damaged interstitial cells. Cardiogenic shock clinically imitated fulminant myocarditis and was managed in accordance with standard protocols [65].

In a cohort study comprising 100 recovered COVID-19 patients, cardiac MRI revealed that 78% had cardiac involvement, with continuous myocardial inflammation present in 60%. This occurrence was found to be independent of pre-existing health conditions, the severity and overall course of the acute illness, and the time elapsed since the initial diagnosis. Eligibility for participation in this study was determined by a period of at least two weeks following the original diagnosis, during which respiratory symptoms cleared up and swab test results came back negative. Compared to control groups, patients who had recovered from COVID-19 exhibited a decreased LVEF and right ventricular ejection fraction (RVEF) while simultaneously having an increased left ventricular volume. These individuals also reported experiencing symptoms such as atypical chest pain, palpitations, dyspnea, and fatigue; 25 patients even noted experiencing these symptoms during activities that would typically cause no discomfort or exhaustion [66].

Another case report discussed the case of a 31-year-old patient with fluctuating symptoms of myopericarditis which was diagnosed based on cardiac MRI and elevated troponin levels, one year after testing positive for COVID-19, thus meeting the criteria of long COVID syndrome. He developed severe chest pain radiating to the scapular region, although no signs of infarction were seen on the ECG. Echocardiogram revealed myopericarditis with an LVEF of 43%. Due to persistent cardiac manifestations like tachycardia, dyspnea, and fatigue, cardiac MRI and scintigraphy were performed. MRI revealed global myocardial hyperintensity. Scintigraphy revealed cold areas, which were suggestive of hyperinflammation [67].

Based on the limited studies available about patients suffering from myocarditis as a sequelae of long COVID syndrome, the common clinical manifestations that the patients presented with were dyspnea, chest pain, palpitations, and a persistent cough. It is noteworthy to mention that general exhaustion remained the most common symptom reported across all the studies.

Diagnosis and investigations 

The diagnosis of myocarditis heavily relies on clinical features [68]. The symptoms associated with SARS-CoV-2-induced myocarditis can vary considerably among patients. Although some may experience mild signs such as shortness of breath and fatigue, others may feel chest pain or tightness upon exertion [2,69,70]. In more severe cases, there may be a deterioration in the condition characterized by tachycardia and indications of acute heart failure such as cardiogenic shock. Additionally, the development of right-sided heart failure symptoms like peripheral edema, elevated jugular venous pressure, and right upper quadrant discomfort has been observed [71]. Fulminant myocarditis emerges within two to three weeks of viral exposure and is considered the most severe form [72]. Early signs of fulminant myocarditis are similar to those of sepsis, including fever, low pulse pressure, cold or patchy extremities, and sinus congestion. As a result, it is crucial for healthcare providers to recognize the various clinical presentations associated with myocarditis promptly. They should conduct a comprehensive assessment of patients who present with potential symptoms to guarantee a timely diagnosis and appropriate management.

Inflammatory and other biomarkers

When a patient is suspected of having myocarditis, several blood tests are performed to determine the levels of different markers, such as lactate and inflammatory markers like CRP, erythrocyte sedimentation rate (ESR), and procalcitonin. These levels are often elevated in myocarditis patients, which could be indicative of an infection. During diagnosis, it's crucial to differentiate between fulminant myocarditis and sepsis because the fluid resuscitation protocol that is commonly used in treating sepsis can lead to fluid overload, worsening the condition in fulminant myocarditis patients. Cardiac enzyme levels like cardiac troponin I, cardiac troponin T, N-terminal pro-B-type natriuretic peptide (NT-proBNP), and BNP should be evaluated when a patient is admitted to the hospital since they're typically increased in acute myocardial harm caused by myocarditis with possible ventricular dilation. Recent studies [69,70,71] have found that COVID-19-infected individuals with myocarditis also have increased levels of troponin and NT-proBNP. Although a negative troponin result may not completely eliminate the possibility of being diagnosed with acute myocarditis, it can still be useful during the acute phase; especially if serial high-sensitivity cardiac troponin tests are negative, since this significantly lowers the probability of diagnosing acute myocarditis. Furthermore, increases in NT-proBNP levels may also occur in COVID-19-infected patients due to potential side effects from severe respiratory illnesses leading to myocardial stress [73].

Cardiac tests

Myocarditis may exhibit ECG irregularities that are frequently linked with pericarditis, such as ST elevation and PR depression. However, these findings are not reliable enough to diagnose the condition and should not be solely relied upon to rule it out. A COVID-19-related myocarditis case serves as an example where neither PR depression nor ST elevation was evident [69]. Myocarditis can also cause other ECG abnormalities, such as new-onset bundle branch block, bradyarrhythmia with advanced atrioventricular nodal block, QT prolongation, pseudo infarct pattern, and premature ventricular complexes [68].

Cardiac MRI is considered the optimal noninvasive diagnostic test due to its high specificity in detecting myocarditis as well as its superior sensitivity of 75%. It is recommended to use the Lake Louis standards when analyzing cardiac MRI images. Previous research is in favor of this approach [52,74]. The criteria proposed involves the integration of T2-weighted images with early and late gadolinium enhancement in order to detect the presence of myocardial edema, hyperemia, myocardial necrosis, and myocardial fibrosis. One limitation of cardiac MRI is its inability to differentiate between inflammation caused by an autoimmune response to the virus and inflammation resulting from a viral infection of the heart, as noted in reference 74. Furthermore, the utilization of cardiac MRI may be limited in individuals who present with significant myocarditis resulting in cardiogenic shock or unstable hemodynamics [75]. Additionally, these individuals may require mechanical ventilation or experience tachyarrhythmias. Under the given circumstances, it may be deemed more advantageous.

Prognosis and outcomes 

The occurrence of myocarditis among individuals who contract COVID-19 is associated with a less favorable prognosis regarding the progression and resolution of the COVID-19 illness. However, the extended-term outlook for mild instances of myocarditis remains uncertain and requires further investigation [68,76]. Researchers indicate that target tissue fibrosis and microangiopathy may result from immune activation and dysfunction, having long-lasting impacts on the patients who are impacted. As a result of worries that COVID-19 could cause myocardial damage, researchers began imaging survivors of the virus using cardiac MRI. They observed a significantly elevated occurrence rate (as high as 78%) of anomalies in patients who underwent cardiac MRI testing within the time frame of 37-71 days following their COVID-19 diagnosis [77]. As per a study conducted by German researchers, a significant proportion of individuals who survived the SARS-CoV-2 infection experienced persistent cardiac involvement even after two months of testing positive. Specifically, 78% of the survivors showed evidence of ongoing cardiac involvement, and 60% continued to display symptoms of myocarditis [14]. These individuals are at risk of developing chronic heart failure. Therefore, it is critical to determine individuals with cardiac involvement as soon as possible so that they can receive cardioprotective therapy and the proper follow-up measures. Nevertheless, similar complications did not occur in patients who had been vaccinated for COVID-19 [78].

Myocarditis and long COVID management and treatment

Myocarditis management and treatment in long COVID syndrome depends on the severity of symptoms, complication, onset, and hemodynamic stability vs. unstability.

Considerable uncertainty surrounds the COVID-19 infection. Similar to other conditions involving acute cardiac injury, the response is expected to vary significantly depending on various factors such as the type of myocardial injury mechanism, severity of the acute illness, therapeutic intervention administered, hemodynamic response, host characteristics, immune-mediated factors, as well as post-treatment care and monitoring [79].

It is recommended that patients experiencing heart failure as a result of COVID-19 myocarditis be treated in accordance with established medical guidelines. This involves the administration of ACE inhibitors, angiotensin receptor blockers (ARBs), angiotensin receptor-neprilysin inhibitors (ARNi), β-blockers, and diuretics [80]. The primary apprehension was that administering ACE inhibitors, ARBs, and ARNIs as a therapeutic intervention for COVID-19 patients could potentially result in unfavorable clinical outcomes owing to the pharmacological mechanisms of these medications. Nevertheless, recent observational studies have indicated that there is no statistically significant distinction in the treatment of patients with ACEi or ARB medication when compared to the cessation of such medications. Therefore, it is commonly advised to commence or persist with these medications throughout and after the illness [81,82].

The recommended course of action for managing patients diagnosed with fulminant myocarditis and experiencing cardiogenic shock involves the administration of inotropes and/or vasopressors during the acute phase, as well as mechanical circulatory support for longer-term management [83-87].

It is crucial to efficiently manage cardiac arrhythmias that are commonly linked with COVID-19 myocarditis, in order to minimize any negative health outcomes for patients. Temporary cardiac pacing may be necessary in instances of bradyarrhythmia, while tachyarrhythmias may be treated effectively with antiarrhythmic medications. In cases where patients are hemodynamically stable, the administration of β-blockers may be deemed appropriate. Conversely, critically ill patients are typically prescribed amiodarone. It is important to acknowledge that the use of amiodarone can result in QTc prolongation, particularly when used in conjunction with azithromycin or hydroxychloroquine, as noted in sources [86-88]. An alternative therapeutic approach may encompass the administration of either lidocaine infusions or oral flecainide [84-86]. In addition, there exist therapeutic options for the SARS-CoV-2 virus that require examination.

Some researchers have suggested the use of anti-inflammatory and anti-cytokine medications, such as IV immunoglobulin (IVIG) and high-dose steroids, due to the potential role of cytokine release syndrome in COVID-19 myocarditis. This recommendation is based on their investigation of the condition. The administration of high doses of steroids to COVID-19 patients has yielded inconsistent results. One retrospective study has shown an improvement in survival rates, while another trial has revealed a decrease in viral clearance and an increased vulnerability to secondary infections and mortality from all causes. These findings have been documented in multiple publications [87-89]. In general, the administration of corticosteroids to COVID-19 patients who were hospitalized was found to be advantageous solely for those who were undergoing invasive mechanical ventilation and oxygen therapy [90].

There are current debates surrounding the administration of renin-angiotensin-aldosterone system (RAAS) antagonists in individuals diagnosed with COVID-19. This is due to the virus's ability to attach to ACE2 receptors during cellular invasion. There is a debate among experts regarding the potential clinical advantages of inhibiting the RAAS, as some argue in favor of it while others have reservations about the possible upregulation of ACE2 that may result from such blockades. The present clinical data substantiate the recommendation of maintaining the RAAS antagonist treatment plan for authorized indications despite the occurrence of COVID-19 infection in patients at a later stage. This approach is endorsed by the Heart Failure Society of America, the American College of Cardiology, and the American Heart Association (AHA) [91]. In contrast, American Heart Association advise against administering nonsteroidal anti-inflammatory drugs (NSAIDs) to individuals with myocarditis due to their potential to induce renal impairment and sodium retention, which may worsen acute ventricular dysfunction [92].

It is crucial to conduct screening for any remaining cardiac involvement during the period of recovery in order to ascertain the magnitude of enduring cardiac ailments that may have been triggered by the COVID-19 infection. In the event that a considerable proportion of the population is discovered to be impacted, it may be fitting to initiate clinical trials of preventive treatments designed to avert enduring complications. The selection of screening tests for post-COVID-19 myocardial dysfunction and arrhythmias ought to be predicated on their respective categorization and cost-benefit analysis. Our suggestion is to perform routine ECG and echocardiogram assessments, supplemented by potential cardiac monitoring, within two to six months after recovery. However, it is important to note that these examinations may not identify minor clinical irregularities. In cases where initial testing reveals abnormalities or when there is a clinical indication, it is advisable to consider advanced imaging techniques such as MRI with gadolinium enhancement or echocardiographic strain. Subsequent research endeavors will elucidate whether a "post-COVID-19 cardiac syndrome" will emerge and the optimal approach for managing patients recuperating from COVID-19's cardiac implications [93].

## Conclusions

Prolonged exposure to COVID-19 can result in serious cardiovascular symptoms such as myocarditis, arrhythmias, and myocardial damage. Due to the wide range of associated symptoms, conducting thorough examinations of patients is crucial while keeping in mind the varying ways in which they may manifest. Inflammatory markers present in blood tests have been found to be closely linked with illness severity, but negative troponin outcomes cannot completely rule out myocarditis. However, consistent high-sensitivity cardiac troponin tests are beneficial for identifying this disease in its early stages. Cardiac MRI is currently the gold standard diagnostic test for myocarditis since it can detect edema, hyperemia, necrosis, and fibrosis in the heart muscle. Nevertheless, cardiac MRI cannot distinguish between viral infection-induced or immune system-driven inflammation.

Patients who develop myocarditis after contracting long COVID have an unfavorable prognosis due to immune system dysfunction that causes irreparable tissue damage through fibrosis and microangiopathy. It is therefore imperative to identify cardiac involvement early on so that affected patients may receive cardioprotective medication and monitoring as necessary. The enhancement of treatment for long COVID syndrome patients experiencing cardiac issues can be facilitated through collaborative efforts among experts from diverse fields, such as pulmonology, cardiology, and infectious disease. Nonetheless, the comprehension of myocarditis or myocardial injury's underlying mechanisms in long COVID syndrome patients and the identification of potential therapeutic targets necessitate further investigation. The execution of initiatives to promote COVID-19 vaccination and preventive measures holds promise in mitigating the incidence of long COVID syndrome and its accompanying cardiac complexities in public health. This approach could assist in averting and advancing the early management of cardiac complications, resulting in improved outcomes for survivors impacted by COVID-19.

## References

[1] Internet Internet (2023). COVID-19 Regional Reports and Updates. https://iris.paho.org/handle/10665.2/53084.

[2] Siripanthong B, Nazarian S, Muser D (2020). Recognizing COVID-19-related myocarditis: the possible pathophysiology and proposed guideline for diagnosis and management. Heart Rhythm.

[3] Agdamag AC, Edmiston JB, Charpentier V (2020). Update on COVID-19 myocarditis. Medicina (Kaunas).

[4] Szarpak L, Pruc M, Filipiak KJ (2022). Myocarditis: a complication of COVID-19 and long-COVID-19 syndrome as a serious threat in modern cardiology. Cardiol J.

[5] Mele D, Flamigni F, Rapezzi C, Ferrari R (2021). Myocarditis in COVID-19 patients: current problems. Intern Emerg Med.

[6] Soriano JB, Murthy S, Marshall JC, Relan P, Diaz JV (2022). A clinical case definition of post-COVID-19 condition by a Delphi consensus. Lancet Infect Dis.

[7] Raman B, Bluemke DA, Lüscher TF, Neubauer S (2022). Long COVID: post-acute sequelae of COVID-19 with a cardiovascular focus. Eur Heart J.

[8] Chilazi M, Duffy EY, Thakkar A, Michos ED (2021). COVID and cardiovascular disease: what we know in 2021. Curr Atheroscler Rep.

[9] (2023). Long COVID or Post-COVID Conditions. https://www.cdc.gov/coronavirus/2019-ncov/long-term-effects/index.html.

[10] Callard F, Perego E (2021). How and why patients made long covid. Soc Sci Med.

[11] (2023). A clinical case definition of post COVID-19 condition by a Delphi consensus, 6 October. A Clinical Case Definition of Post Covid-19 Condition by a Delphi Consensus, 6 October 2021.

[12] Logue JK, Franko NM, McCulloch DJ, McDonald D, Magedson A, Wolf CR, Chu HY (2021). Sequelae in adults at 6 months after COVID-19 infection. JAMA Netw Open.

[13] Davis HE, Assaf GS, McCorkell L (2021). Characterizing long COVID in an international cohort: 7 months of symptoms and their impact. EClinicalMedicine.

[14] van der Sar-van der Brugge S, Talman S, Boonman-de Winter L, de Mol M, Hoefman E, van Etten RW, De Backer IC (2021). Pulmonary function and health-related quality of life after COVID-19 pneumonia. Respir Med.

[15] Townsend L, Dyer AH, Jones K (2020). Persistent fatigue following SARS-CoV-2 infection is common and independent of severity of initial infection. PLoS One.

[16] Augustin M, Schommers P, Stecher M (2021). Post-COVID syndrome in non-hospitalised patients with COVID-19: a longitudinal prospective cohort study. Lancet Reg Health Eur.

[17] Puntmann VO, Carerj ML, Wieters I (2020). Outcomes of cardiovascular magnetic resonance imaging in patients recently recovered from coronavirus disease 2019 (COVID-19). JAMA Cardiol.

[18] Xiong Q, Xu M, Li J, Liu Y, Zhang J, Xu Y, Dong W (2021). Clinical sequelae of COVID-19 survivors in Wuhan, China: a single-centre longitudinal study. Clin Microbiol Infect.

[19] Chopra V, Flanders SA, O'Malley M, Malani AN, Prescott HC (2021). Sixty-day outcomes among patients hospitalized with COVID-19. Ann Intern Med.

[20] Taquet M, Dercon Q, Harrison PJ (2022). Six-month sequelae of post-vaccination SARS-CoV-2 infection: a retrospective cohort study of 10,024 breakthrough infections. Brain Behav Immun.

[21] Garrigues E, Janvier P, Kherabi Y (2020). Post-discharge persistent symptoms and health-related quality of life after hospitalization for COVID-19. J Infect.

[22] Carfì A, Bernabei R, Landi F (2020). Persistent symptoms in patients after acute COVID-19. JAMA.

[23] (2023). The Prevalence of Long COVID Symptoms and COVID-19 Complications. https://www.ons.gov.uk/news/statementsandletters/theprevalenceoflongcovidsymptomsandcovid19complications.

[24] (2023). Prevalence of Ongoing Symptoms Following Coronavirus (COVID-19) Infection in the UK: 30 March 2023. https://www.ons.gov.uk/peoplepopulationandcommunity/healthandsocialcare/conditionsanddiseases/bulletins/prevalenceofongoingsymptomsfollowingcoronaviruscovid19infectionintheuk/latest.

[25] (2023). Coronavirus in the UK: Cases in England. https://coronavirus.data.gov.uk/details/cases?areaType=nation&areaName=England.

[26] Klein J, Wood J, Jaycox J (2022). Distinguishing Features of Long COVID Identified Through Immune Profiling. medRxiv.

[27] (2023). WHO Coronavirus (COVID-19) Dashboard. https://covid19.who.int.

[28] Xiong TY, Redwood S, Prendergast B, Chen M (2020). Coronaviruses and the cardiovascular system: acute and long-term implications. Eur Heart J.

[29] Sherif ZA, Gomez CR, Connors TJ, Henrich TJ, Reeves WB (2023). Pathogenic mechanisms of post-acute sequelae of SARS-CoV-2 infection (PASC). Elife.

[30] Silva Andrade B, Siqueira S, de Assis Soares WR (2021). Long-COVID and post-COVID health complications: an up-to-date review on clinical conditions and their possible molecular mechanisms. Viruses.

[31] Dixit NM, Churchill A, Nsair A, Hsu JJ (2021). Post-acute COVID-19 syndrome and the cardiovascular system: what is known?. Am Heart J Plus.

[32] Nalbandian A, Sehgal K, Gupta A (2021). Post-acute COVID-19 syndrome. Nat Med.

[33] Ståhlberg M, Reistam U, Fedorowski A (2021). Post-COVID-19 tachycardia syndrome: a distinct phenotype of post-acute COVID-19 syndrome. Am J Med.

[34] Elseidy SA, Awad AK, Vorla M, Fatima A, Elbadawy MA, Mandal D, Mohamad T (2022). Cardiovascular complications in the post-acute COVID-19 syndrome (PACS). Int J Cardiol Heart Vasc.

[35] Holland DJ, Blazak PL, Martin J, Broom J, Poulter RS, Stanton T (2022). Myocarditis and cardiac complications associated with COVID-19 and mRNA vaccination: a pragmatic narrative review to guide clinical practice. Heart Lung Circ.

[36] Daniels CJ, Rajpal S, Greenshields JT (2021). Prevalence of clinical and subclinical myocarditis in competitive athletes with recent SARS-CoV-2 infection: results from the big ten COVID-19 cardiac registry. JAMA Cardiol.

[37] Theetha Kariyanna P, Sutarjono B, Grewal E (2020). A systematic review of COVID-19 and myocarditis. Am J Med Case Rep.

[38] Blankstein R, Chandrashekhar Y (2021). New insights on COVID-19 and the heart. JACC Cardiovasc Imaging.

[39] Annie FH, Alkhaimy H, Nanjundappa A, Elashery A (2022). Association between myocarditis and mortality in COVID-19 patients in a large registry. Mayo Clin Proc Innov Qual Outcomes.

[40] Hopwood AJ, Jordan-Villegas A, Gutierrez LD (2021). Severe acute respiratory syndrome coronavirus-2 pneumonia in a newborn treated with remdesivir and coronavirus disease 2019 convalescent plasma. J Pediatric Infect Dis Soc.

[41] Sattar Y, Sandhyavenu H, Patel N (2023). In-hospital outcomes of COVID-19 associated myocarditis (from a nationwide inpatient sample database study). Am J Cardiol.

[42] Beşler MS, Arslan H (2020). Acute myocarditis associated with COVID-19 infection. Am J Emerg Med.

[43] Ramli NA, Subramaniam MR, Ramli N, Adam S, Fauzi MF, Loon NK, Sayuti KA (2023). Post Covid-19 with myocarditis: a case report. Int J Cardiol.

[44] Munoz D, Malik H, Eickenhorst D, Newman S, Varughese C, Ali F (2021). Cardiac screening in a young adult male leading to discovery of post-COVID myocarditis with asymptomatic large apical left ventricular thrombus. CASE (Phila).

[45] Pavon AG, Meier D, Samim D (2020). First documentation of persistent SARS-Cov-2 infection presenting with late acute severe myocarditis. Can J Cardiol.

[46] Rivera-Morales M D, Pell R, Rubero J (2020). Acute myopericarditis in the post COVID-19 recovery phase. October 29.

[47] Gyöngyösi M, Alcaide P, Asselbergs FW (2023). Long COVID and the cardiovascular system-elucidating causes and cellular mechanisms in order to develop targeted diagnostic and therapeutic strategies: a joint scientific statement of the ESC working groups on cellular biology of the heart and myocardial and pericardial diseases. Cardiovasc Res.

[48] Khetpal V, Berkowitz J, Vijayakumar S (2022). Long-term cardiovascular manifestations and complications of covid-19: spectrum and approach to diagnosis and management. R I Med J (2013).

[49] Kotecha T, Knight DS, Razvi Y (2021). Patterns of myocardial injury in recovered troponin-positive COVID-19 patients assessed by cardiovascular magnetic resonance. Eur Heart J.

[50] Breitbart P, Koch A, Schmidt M (2021). Clinical and cardiac magnetic resonance findings in post-COVID patients referred for suspected myocarditis. Clin Res Cardiol.

[51] Huang L, Zhao P, Tang D (2020). Cardiac involvement in patients recovered from COVID-2019 identified using magnetic resonance imaging. JACC Cardiovasc Imaging.

[52] Galea N, Marchitelli L, Pambianchi G (2021). T2-mapping increase is the prevalent imaging biomarker of myocardial involvement in active COVID-19: a cardiovascular magnetic resonance study. J Cardiovasc Magn Reson.

[53] Ho JS, Sia CH, Chan MY, Lin W, Wong RC (2020). Coronavirus-induced myocarditis: a meta-summary of cases. Heart Lung.

[54] Leung TY, Chan AY, Chan EW (2020). Short- and potential long-term adverse health outcomes of COVID-19: a rapid review. Emerg Microbes Infect.

[55] Nurek M, Rayner C, Freyer A, Taylor S, Järte L, MacDermott N, Delaney BC (2021). Recommendations for the recognition, diagnosis, and management of long COVID: a Delphi study. Br J Gen Pract.

[56] Sheikh AB, Javed N, Sheikh AA, Upadhyay S, Shekhar R (2021). Diabetes insipidus and concomitant myocarditis: a late sequelae of COVID-19 infection. J Investig Med High Impact Case Rep.

[57] Amin A, Eftekhar SP, Ziaie N (2021). Clinically suspected myocarditis in COVID-19 patients: case series and review of the literature. Clin Case Rep.

[58] Davis MG, Bobba A, Chourasia P (2022). COVID-19 associated myocarditis clinical outcomes among hospitalized patients in the United States: a propensity matched analysis of national inpatient sample. Viruses.

[59] Rathore SS, Rojas GA, Sondhi M (2021). Myocarditis associated with Covid-19 disease: a systematic review of published case reports and case series. Int J Clin Pract.

[60] Cannon HR, Bobba A, Shekhar R (2023). Nationwide analysis of the clinical outcomes of patients admitted with COVID-19 infection with myocarditis and racial disparities in mortality. Curr Probl Cardiol.

[61] Davis MG, Bobba A, Majeed H (2023). COVID-19 with stress cardiomyopathy mortality and outcomes among patients hospitalized in the United States: a propensity matched analysis using the national inpatient sample database. Curr Probl Cardiol.

[62] Çınar T, Hayıroğlu Mİ, Çiçek V, Uzun M, Orhan AL (2020). COVID-19 and acute myocarditis: current literature review and diagnostic challenges. Rev Assoc Med Bras (1992).

[63] Lovell JP, Čiháková D, Gilotra NA (2022). COVID-19 and myocarditis: review of clinical presentations, pathogenesis and management. Heart Int.

[64] Shrestha AB, Mehta A, Pokharel P (2023). Long COVID syndrome and cardiovascular manifestations: a systematic review and meta-analysis. Diagnostics (Basel).

[65] Tavazzi G, Pellegrini C, Maurelli M (2020). Myocardial localization of coronavirus in COVID-19 cardiogenic shock. Eur J Heart Fail.

[66] Ghafoor N, Islam MM, Shakil SN (2022). Observation of myocardial involvement in patients recovered from COVID-19 by using cardiac magnetic resonance imaging, in a tertiary care hospital, Bangladesh. Mymensingh Med J.

[67] Vera-Lastra O, Lucas-Hernández A, Ruiz-Montiel JE, Gonzalez-Rodriguez VR, Pineda-Galindo LF (2021). Myopericarditis as a manifestation of long COVID syndrome. Cureus.

[68] Zeng JH, Liu YX, Yuan J (2020). First case of COVID-19 complicated with fulminant myocarditis: a case report and insights. Infection.

[69] Inciardi RM, Lupi L, Zaccone G (2020). Cardiac involvement in a patient with coronavirus disease 2019 (COVID-19). JAMA Cardiol.

[70] Kim IC, Kim JY, Kim HA, Han S (2020). COVID-19-related myocarditis in a 21-year-old female patient. Eur Heart J.

[71] Kociol RD, Cooper LT, Fang JC (2020). Recognition and initial management of fulminant myocarditis: a scientific statement from the American Heart Association. Circulation.

[72] Esfandiarei M, McManus BM (2008). Molecular biology and pathogenesis of viral myocarditis. Annu Rev Pathol.

[73] Januzzi J.L (2020). Troponin and BNP use in COVID-19. March.

[74] Ferreira VM, Schulz-Menger J, Holmvang G (2018). Cardiovascular magnetic resonance in nonischemic myocardial inflammation: expert recommendations. J Am Coll Cardiol.

[75] Leone O, Veinot JP, Angelini A (2012). 2011 consensus statement on endomyocardial biopsy from the Association for European Cardiovascular Pathology and the Society for Cardiovascular Pathology. Cardiovasc Pathol.

[76] Ali M, Shiwani HA, Elfaki MY (2022). COVID-19 and myocarditis: a review of literature. Egypt Heart J.

[77] Atri L, Morgan M, Harrell S (2021). Role of cardiac magnetic resonance imaging in the diagnosis and management of COVID-19 related myocarditis: clinical and imaging considerations. World J Radiol.

[78] Khanna S, Amarasekera AT, Li C (2022). The utility of cardiac magnetic resonance imaging in the diagnosis of adult patients with acute myocarditis: a systematic review and meta-analysis. Int J Cardiol.

[79] Kornowski R, Witberg G (2022). Acute myocarditis caused by COVID-19 disease and following COVID-19 vaccination. Open Heart.

[80] Mitrani RD, Dabas N, Goldberger JJ (2020). COVID-19 cardiac injury: implications for long-term surveillance and outcomes in survivors. Heart Rhythm.

[81] McDonagh TA, Metra M, Adamo M (2021). 2021 ESC guidelines for the diagnosis and treatment of acute and chronic heart failure. Eur Heart J.

[82] Lopes RD, Macedo AV, de Barros E Silva PG (2021). Effect of discontinuing vs continuing angiotensin-converting enzyme inhibitors and angiotensin ii receptor blockers on days alive and out of the hospital in patients admitted with COVID-19: a randomized clinical trial. JAMA.

[83] Mehra MR, Desai SS, Kuy S, Henry TD, Patel AN (2020). Cardiovascular disease, drug therapy, and mortality in Covid-19. N Engl J Med.

[84] Frustaci A, Morgante E, Antuzzi D, Russo MA, Chimenti C (2012). Inhibition of cardiomyocyte lysosomal activity in hydroxychloroquine cardiomyopathy. Int J Cardiol.

[85] Lavalle C, Trivigno S, Vetta G (2021). Flecainide in ventricular rrhythmias: from old myths to new perspectives. J Clin Med.

[86] Della Rocca DG, Tarantino N, Trivedi C (2020). Non-pulmonary vein triggers in nonparoxysmal atrial fibrillation: Implications of pathophysiology for catheter ablation. J Cardiovasc Electrophysiol.

[87] Mehta P, McAuley DF, Brown M, Sanchez E, Tattersall RS, Manson JJ (2020). COVID-19: consider cytokine storm syndromes and immunosuppression. Lancet.

[88] Wu C, Chen X, Cai Y (2020). Risk factors associated with acute respiratory distress syndrome and death in patients with coronavirus disease 2019 pneumonia in Wuhan, China. JAMA Intern Med.

[89] Russell CD, Millar JE, Baillie JK (2020). Clinical evidence does not support corticosteroid treatment for 2019-nCoV lung injury. Lancet.

[90] Frustaci A, Chimenti C (2015). Immunosuppressive therapy in myocarditis. Circ J.

[91] Friedrich MG, Sechtem U, Schulz-Menger J (2009). Cardiovascular magnetic resonance in myocarditis: a JACC white paper. J Am Coll Cardiol.

[92] Ojha V, Verma M, Pandey NN (2021). Cardiac magnetic resonance imaging in coronavirus disease 2019 (COVID-19): a systematic review of cardiac magnetic resonance imaging findings in 199 patients. J Thorac Imaging.

[93] Chimenti C, Magnocavallo M, Ballatore F (2022). Prevalence and clinical implications of COVID-19 myocarditis. Card Electrophysiol Clin.

